# Successful Obliteration of a Pseudoaneurysm from Post-CEA Repair Secondary to a Pruitt-Inahara Shunt Using a Stent Graft

**DOI:** 10.1155/2013/382485

**Published:** 2013-07-31

**Authors:** Vishal Dahya, Prasad Chalasani

**Affiliations:** Florida State University College of Medicine, 1115 West Call Street, Tallahassee, FL 32306-4300, USA

## Abstract

Pseudoaneurysms of the carotid artery are very uncommon complications following carotid endarterectomy. Pseudoaneurysms are usually caused by any kind of blunt injury or trauma during carotid artery surgery. CEA has become an increasingly more common vascular surgery performed in the United States. The standard of treatment for a carotid PA has been open surgical repair with excision of the defect and then a graft reconstruction of the artery. Advancements in endovascular intervention have helped to make it a more popular choice in treatment because of the positive results and less invasive approach. This case report describes the successful obliteration of a large post-CEA PA using a stent graft. The PA was likely secondary to the use of a Pruitt-lnahara Shunt because it was found to be distal to the endarterectomized area of the carotid artery which means that the defect was likely caused by the balloon portion of the shunt. This case demonstrates the feasibility of using endovascular interventional techniques to treat a PA using a stent graft.

## 1. Introduction

Pseudoaneurysms (PAs) of the carotid artery are very uncommon complications following carotid endarterectomy (CEA) [1, 2]. PAs are usually caused by any kind of blunt injury or trauma during carotid artery surgery [3]. CEA has become an increasingly more common vascular surgery performed in the United States. More common complications of this type of surgery include hematoma formation, stroke, myocardial infarction, and cranial nerve injury [2]. The incidence of a post-CEA PA is estimated to be around 0.3%, and it has been suggested by various researchers that the risk of this complication is increased by the use of patch closure [4–6]. The standard of treatment for a carotid PA has been open surgical repair with excision of the defect and then a graft reconstruction of the artery. Advancements in endovascular intervention have helped to make it a more popular choice in treatment because of the positive results and less invasive approach [7, 8]. This case report describes the successful obliteration of a large post-CEA PA using a stent graft. The PA was likely secondary to the use of a Pruitt-Inahara Shunt because it was found to be distal to the endarterectomized area of the carotid artery which means that the defect was likely caused by the balloon portion of the shunt. This case demonstrates the feasibility of using endovascular interventional techniques to treat a PA using a stent graft.

## 2. Case Report

**Figure 1:**
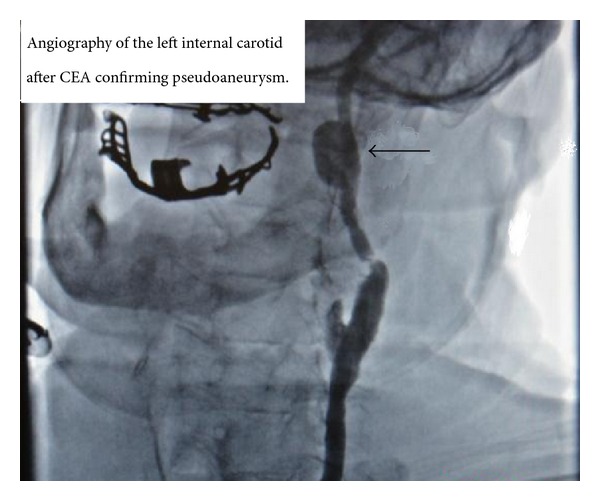
Computed tomography angiography of left internal carotid artery; pseudoaneurysm location is confirmed.

**Figure 2:**
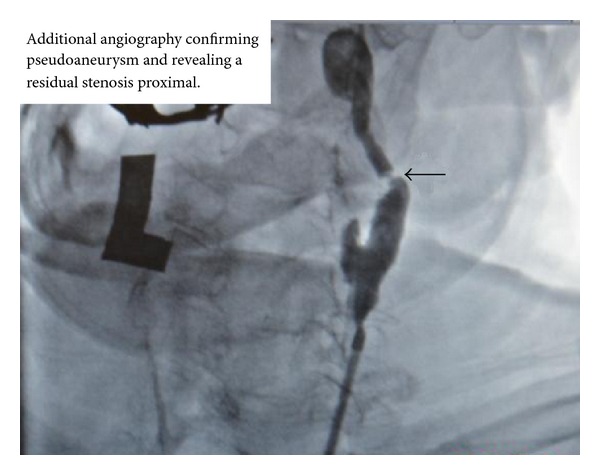
Additional angiography; residual stenosis is seen proximal to the pseudoaneurysm.

**Figure 3:**
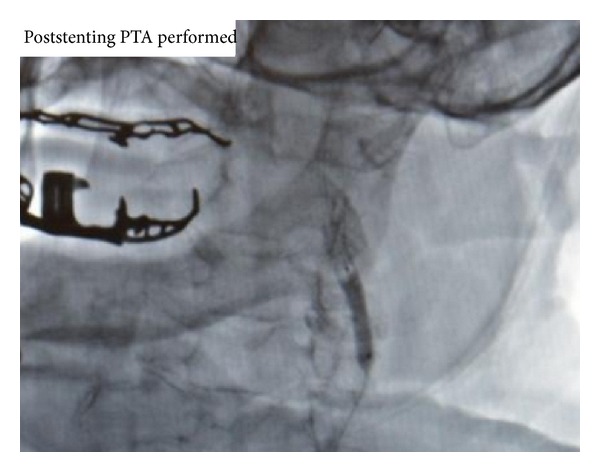
Poststenting percutaneous transluminal angiography; stent graft placement is seen with angiography.

**Figure 4:**
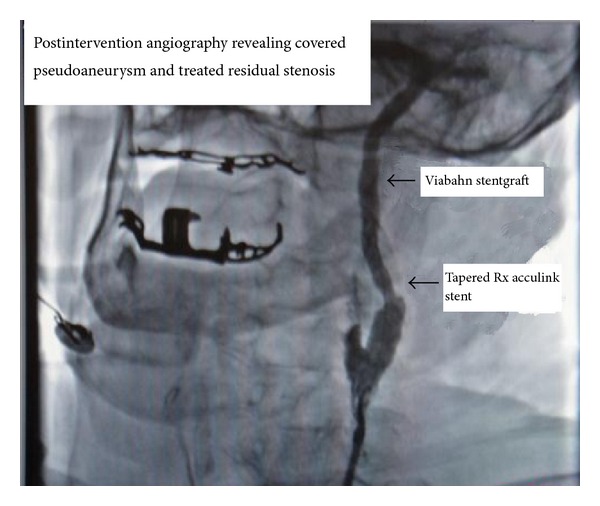
Postintervention angiography; pseudoaneurysm is successfully obliterated and residual stenosis has been treated.

An 86-year-old man presented with a headache and blurred vision. His surgical history was significant for an uneventful left sided CEA with a Vascular Patch (Synovis VG-0106N) and utilizing a Pruitt-Inahara Shunt during the procedure. This surgery was performed 2 months ago for high grade left internal carotid stenosis (90%). Patient was having no other associated symptoms and had no clinical signs of infection. Computed tomography (CT) angiography of the head and neck was then performed, revealing a 1.2 × 2.0 cm pseudoaneurysm with a small amount of surrounding thrombus within the distal left cervical internal carotid artery just proximal to the petrous portion (Figure 1). The PA was found to be distal to the endarterectomized area of the carotid artery which means that the defect was likely caused by the balloon portion of the shunt. A short segment of high grade stenosis (80%) was also revealed in the postbulbar region of the proximal left internal carotid artery (Figure 2). Patient was then taken to the catheterization lab for endovascular exclusion of the PA. The right groin was prepped, and a 6 French catheter was placed in the femoral artery. An internal mammary catheter was used and then selectively cannulated the internal mammary to gain access to the left carotid artery where angiography was performed. A 0.035 wire was then passed through, and a 7 French 9 cm long sheath was passed into the left common carotid artery where a repeat angiography was performed. Then a 0.014 BMW wire was passed across those lesions, and the position was confirmed. A 6 × 50 mm long covered Viabahn stent (W. L. Gore & Associates Inc., Flagstaff, AZ) was successfully deployed across the PA, but there were still the tight stenosis and 90-degree bend of the graft anastomosis of the endarterectomy site. For the residual stenosis, a 7 × 10 stent (Nitinol Acculink) was successfully deployed and expanded using a 5 Balloon to 6 Atmospheres (Figure 3). After full expansion was noted, the balloon was withdrawn, and completion angiography was performed confirming complete obliteration of the PA (Figure 4). Patient recovered with no postoperative neurological symptoms and had no complications upon 3-year followup.

## 3. Discussion

Pseudoaneurysms are known to be a rare postoperative complication of CEA, but research is being done to identify the exact cause of these defects. Reported causes of post-CEA PAs include infection of prosthetic material, blunt injury, and suture failure [1, 2]. Not many cases have been reported of PAs being caused by the shunt used during the CEA procedure. The Pruitt Inahara Shunt is a device that allows for the maintenance of cerebral blood flow during carotid surgery. This shunt is placed into the vessel and uses balloons to keep the artery patent as the plaque is being removed [9]. Since the PA in our case was located distal to the vascular patch site or the endarterectomized area of the carotid artery, we concluded that the balloon portion of the shunt likely caused blunt trauma to the artery. This blunt trauma allowed for the formation of the left internal carotid PA [10]. The standard treatment of PAs for many years has been open carotid surgery, but endovascular intervention has made progress which has enabled it to gain more popularity [7, 8]. In our case, endovascular intervention was chosen because of the large size of the PA and the history of prior CEA which would increase the difficulty of an open repair. A self-expanding stent graft was also chosen to allow for complete obliteration of the PA and improve the carotid blood flow.

## 4. Conclusion

In conclusion, endovascular treatment of a post-CEA PA with a stent graft has shown encouraging results, but long term data is needed to make a definitive decision that this is the therapy of choice. A similar case was described by the Department of Vascular Surgery at the University of Florence. This study concluded with the similar notion that PA formation can be seen with overinflation of the balloon [11]. We cannot be certain of the exact mechanism of how the PA formed, and more studies must be done to identify the complications of shunt induced blunt trauma. A larger case series is also needed on post-CEA PAs to understand the exact genesis and formation of the PA.
